# Characterization of Head Transcriptome and Analysis of Gene Expression Involved in Caste Differentiation and Aggression in *Odontotermes formosanus* (Shiraki)

**DOI:** 10.1371/journal.pone.0050383

**Published:** 2012-11-29

**Authors:** Qiuying Huang, Pengdong Sun, Xuguo Zhou, Chaoliang Lei

**Affiliations:** 1 Hubei Insect Resources Utilization and Sustainable Pest Management Key Laboratory, Huazhong Agricultural University, Wuhan, China; 2 Department of Entomology, S-225 Agricultural Science Center N, University of Kentucky, Lexington, Kentucky, United States of America; Auburn University, United States of America

## Abstract

**Background:**

The subterranean termite *Odontotermes formosanus* (Shiraki) is a serious insect pest of trees and dams in China. To date, very little is known about genomic or transcriptomic data for caste differentiation and aggression in *O. formosanus*. Hence, studies on transcriptome and gene expression profiling are helpful to better understand molecular basis underlying caste differentiation and aggressive behavior in *O. formosanus*.

**Methodology and Principal Findings:**

Using the Illumina sequencing, we obtained more than 57 million sequencing reads derived from the heads of *O. formosanus*. These reads were assembled into 116,885 unique sequences (mean size  =  536 bp). Of the unigenes, 30,646 (26.22%) had significant similarity with proteins in the NCBI nonredundant protein database and Swiss-Prot database (E-value<10^−5^). Of these annotated unigenes, 10,409 and 9,009 unigenes were assigned to gene ontology categories and clusters of orthologous groups, respectively. In total, 19,611 (25.52%) unigenes were mapped onto 242 pathways using the Kyoto Encyclopedia of Genes and Genomes Pathway database (KEGG). A total of 11,661 simple sequence repeats (SSRs) were predicted from the current transcriptome database. Moreover, we detected seven putative genes involved in caste differentiation and six putative genes involved in aggression. The qPCR analysis showed that there were significant differences in the expression levels of the three putative genes *hexamerin 2*, *β-glycosidase* and *bicaudal D* involved in caste differentiation and one putative gene *Cyp6a20* involved in aggression among workers, soldiers and larvae of *O. formosanus*.

**Conclusions:**

To our knowledge, this is the first study to characterize the complete head transcriptome of a higher fungus-cultivating termite using high-throughput sequencing. Our study has provided the comprehensive sequence resources available for elucidating molecular basis underlying caste differentiation and aggressive behavior in *O. formosanus*.

## Introduction

Termites are a group of eusocial insects of immense ecological and economical importance. In recent years, studies of genomics and gene expression in termites have attracted increasing interest [1]–[5]. Advances on functional genomics research in termites are helpful to better understand unique and interesting features of termite biology [6], such as understanding molecular basis of aggression and caste differentiation in termites [7].

The subterranean termite, *Odontotermes formosanus* (Shiraki) (Isoptera: Termitidae), is a higher fungus-cultivating termite that distributes throughout Southeast Asia, including China, Burma, India, Japan, Thailand, and Vietnam [8]. This termite species is an important pest of crops, plantations, and forests in China. Furthermore, this species can build large subterranean cavities inside earthen dikes and dams, thereby damaging piping, which can result in the collapse of the dikes and dams [9]. To date, the patterns of caste differentiation and intercolonial aggression in *O. formosanus* have been studied [10]–[12], but there are no research reports about molecular basis underlying its caste differentiation and aggression. Despite its significant importance of biology and economics, genomic sequence resources available for *O. formosanus* are very scarce. Up to June 28th, 2012, we found that there are about 140,730 ESTs and 26,207 nucleotide sequences in NCBI databases for *Coptotermes*, followed by *Reticulitermes* (24,681 ESTs and 4,664 nucleotide sequences), *Macrotermes* (1,708 ESTs and 822 nucleotide sequences) and *Cryptotermes* (3 ESTs and 323 nucleotide sequences). However, there are no ESTs and only 818 nucleotide sequences deposited in NCBI databases for *Odontotermes*. Therefore, application of the advanced sequencing technology to characterize transcriptome and obtain more ESTs of *Odontotermes* is very necessary.

Currently, some advanced sequencing technologies, such as Illumina sequencing and 454 pyrosequencing, have been used to carry out high-throughput sequencing and have rapidly improved the efficiency and speed of mining genes [13]–[18]. Moreover, these sequencing technologies have greatly improved the sensitivity of gene expression profiling, and is expected to promote collaborative and comparative genomics studies [19], [20]. Thus, we selected the Illumina sequencing to characterize the complete head transcriptome of *O. formosanus*.

In the present study, a total of 57,271,634 raw sequencing reads were generated from one plate (8 lanes) of sequencing. After transcriptome assembly, 221,728 contigs were obtained, and these contigs were further clustered into 116,885 unigenes with 9,040 distinct clusters and 107,845 distinct singletons. In the head transcriptome database, we predicted simple sequence repeats (SSRs), and detected putative genes involved in caste differentiation and aggression. Furthermore, we compared the gene expression profiles of the three putative genes involved in caste differentiation and one putative gene involved in aggression among workers, soldiers and larvae of *O. formosanus*. The assembled, annotated transcriptome sequences and gene expression profiles provide an invaluable resource for the identification of genes involved in caste differentiation, aggressive behavior and other biological characters in *O. formosanus* and other termite species.

## Results and Discussion

### Illumina Paired-end Sequencing and *de novo* Assemble

**Figure 1:**
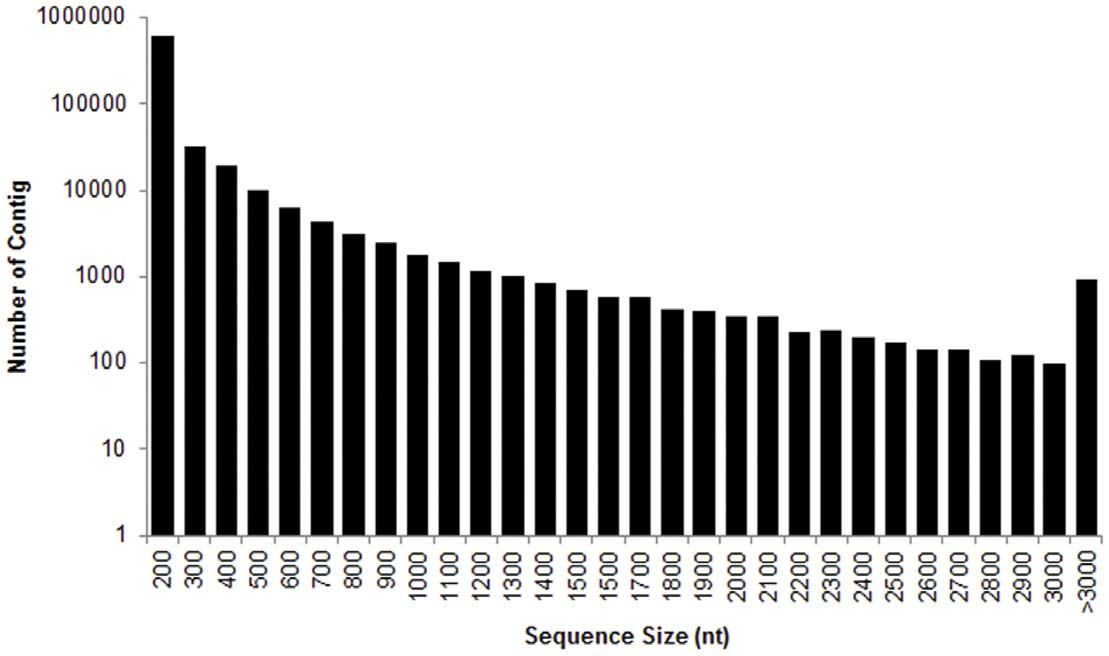
Length distribution of *Odontotermes formosanus* contigs. Histogram presentation of sequence-length distribution for significant matches that was found. The x-axis indicates sequence sizes from 200 nt to >3000 nt. The y-axis indicates the number of contigs for every given size.

**Figure 2:**
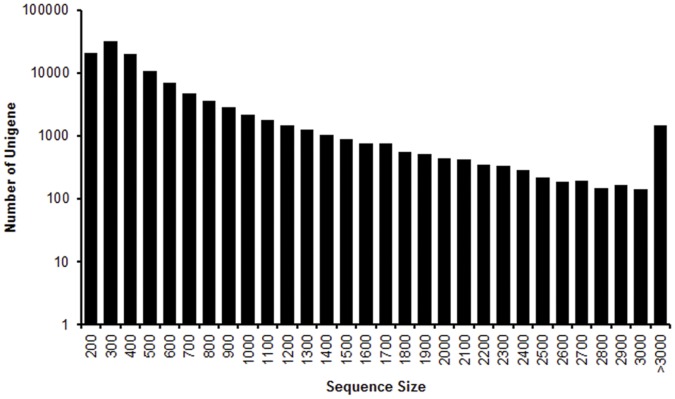
Length distribution of *Odontotermes formosanus* unigenes. Histogram presentation of sequence-length distribution for significant matches that was found. The x-axis indicates sequence sizes from 200 nt to >3000 nt. The y-axis indicates the number of uingenes for every given size. The results of sequence-length matches (with a cut-off E-value of 1.0E-5) in the nr databases are greater among the longer assembled sequences.

Total RNA was extracted from the worker heads of the different colonies. Using Illumina paired-end sequencing technology, a total of 57,271,634 raw sequencing reads were generated from a 200 bp insert library. An assembler, Trinity was employed for *de novo* assembly [21]. After stringent quality check and data cleaning, approximately 54 million high-quality reads were obtained with 98.09% Q_20_ bases (base quality more than 20). Based on the high quality reads, a total of 221,728 contigs were assembled with an average length of 302 bp. The size distribution of these contigs is shown in Figure 1. Then the reads were mapped back to contigs, with paired-end reads we were able to detect contigs from the same transcript as well as the distances between these contigs. After clustering these unigenes using TGICL software [22], contigs can finally generate 116,885 unigenes with 9,040 distinct clusters and 107,845 distinct singletons (Table 1). The length of assembled unigenes ranged from 150 to 17,355 bp. There were 83,002 unigenes (71.01%) with length varying from 150 to 500 bp, 26,916 unigenes (23.03%) in the length range of 501 to 1500 bp, and 6967 unigenes (5.96%) with length more than 1500 bp. The size distribution of these unigenes is showed in Figure 2.

**Table 1:** Summary of the head transcriptome of *Odontotermes formosanus*.

Total raw reads	57,271,634
Total clean reads	53,477,764
Total clean nucleotides (nt)	4,812,998,760
GC percentage	42.80%
Total number of contigs	221,728
Mean length of contigs	302 bp
Total number of unigenes	116,885
Mean length of unigenes	536 bp
Distinct clusters	9,040
Distinct singletons	107,845
Q20 percentage	98.09%

### Functional Annotation by Searching Against Public Databases

**Figure 3:**
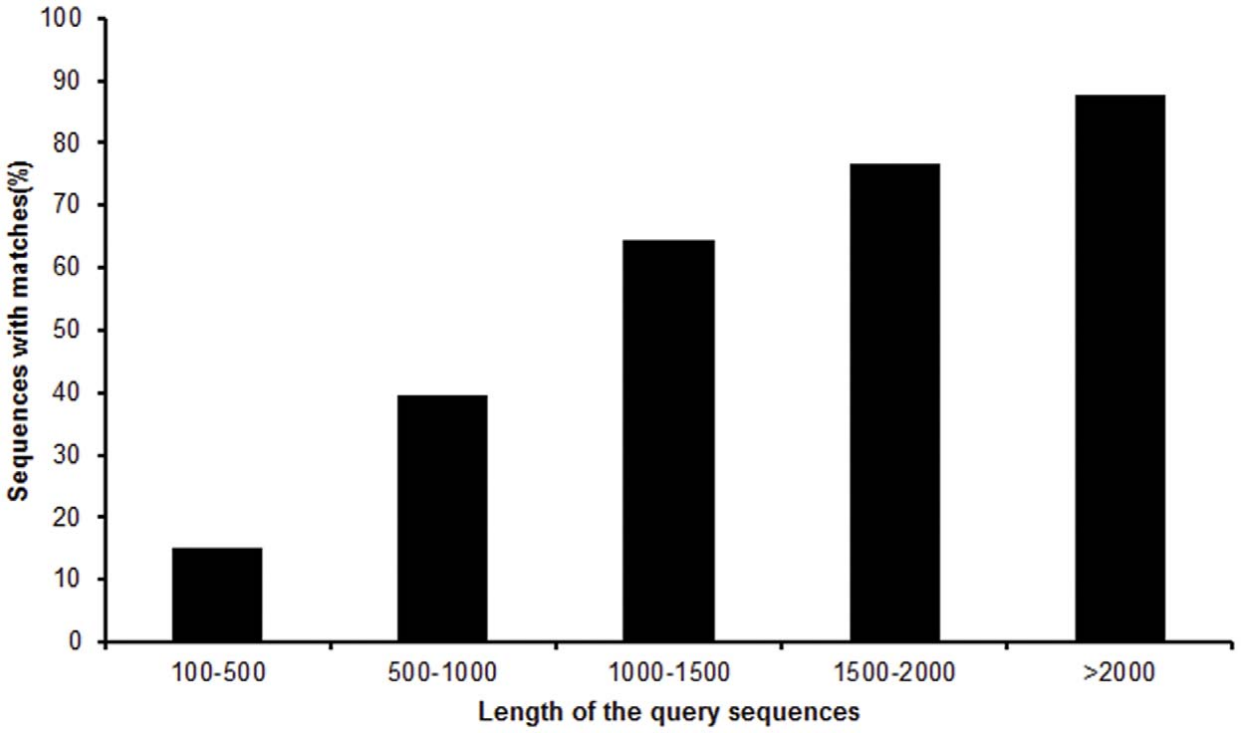
Effect of query sequence length on the percentage of sequences with significant matches. The proportion of sequences with matches (with a cut-off E-value of 1.0E-5) in nr database is greater among the longer assembled sequences.

**Figure 4:**
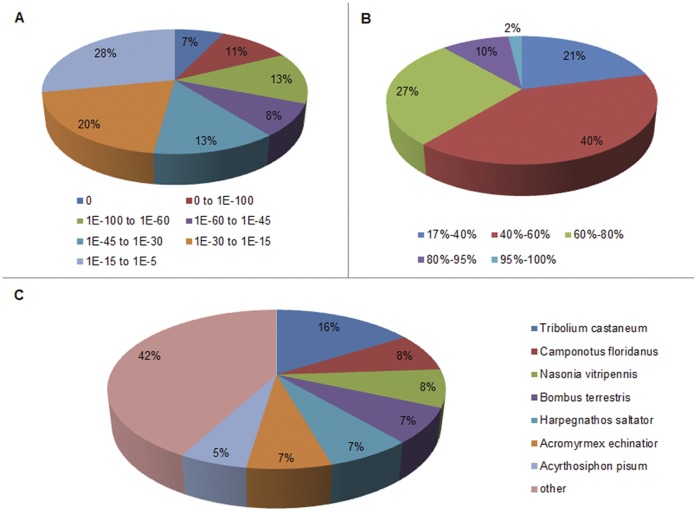
Characteristics of homology search of Illumina sequences against the nr database. (A) E-value distribution of BLAST hits for each unique sequence with a cut-off E-value of 1.0E-5. (B) Similarity distribution of the top BLAST hits for each sequence. (C) Species distribution is shown as a percentage of the total homologous sequences with an E-value of at least 1.0E-5. We used the first hit of each sequence for analysis.

For validation and annotation of assembled unigenes, sequence similarity search was conducted against NCBI non-redundant protein (nr) database and Swiss-Prot protein database using BLASTX algorithm with an E-value threshold of 10^−5^. By this approach, out of 116,885 unigenes, 30,427 genes (26.03% of all distinct sequences) returned an above cut-off BLAST result (Table S1). Because of the relatively short length of distinct gene sequences and lacking genome information in *O. formosanus*, most of the 86,459 assembled sequences could not be matched to known genes (73.97%). Figure 3 indicates that the percentage of matched sequences in nr databases increased as assembled sequences got longer. Specifically, an 87.77% of match efficiency was observed for sequences longer than 2,000 bp, whereas the match efficiency decreased to 39.67% for those ranging from 500 to 1,000 bp and to 14.95% for sequences between 100 to 500 bp (Figure 3). The result indicates that the proportion of sequences with matches in the nr database is greater among the longer assembled sequences. The E-value distribution of the top hits in the nr database ranged from 0 to 1.0E^−5^ (Figure 4A). The similarity distribution of the top BLAST hits for each sequence ranged from 17% to 100% (Figure 4B). For species distribution, 16.0% of the distinct sequences have top matches trained with sequences from *Tribolium castaneum* (Figure 4C). Of all the unigenes, 22,895 (19.59%) had BLAST hits in Swiss-Prot database and matched to 12,497 unique protein entries.

### Functional Classification by GO and COG

**Figure 5:**
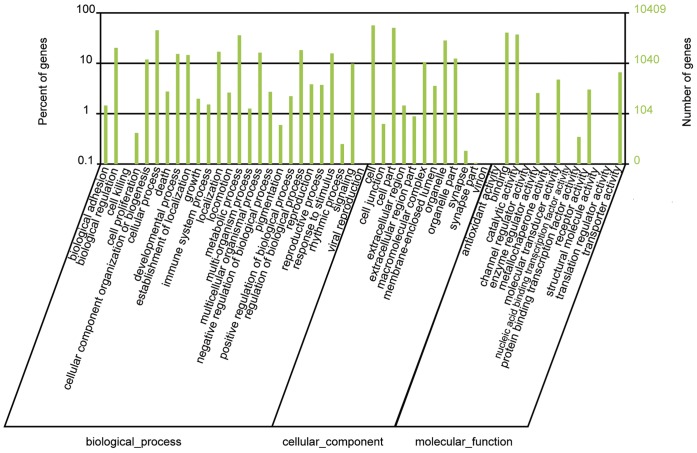
Histogram presentation of Gene Ontology classification. The results are summarized in three main categories: biological process, cellular component and molecular function. The right y-axis indicates the number of genes in a category. The left y-axis indicates the percentage of a specific category of genes in that main category.

GO functional analyses provide GO functional classification annotation [23]. On the basis of nr annotation, the Blast2GO program was used to obtain GO annotation for unigenes [24]. Then the WEGO software was used to perform GO functional classification for these unigenes [25]. In total, 10,409 unigenes with BLAST matches to known proteins were assigned to gene ontology classes with 52,610 functional terms. Of them, assignments to the biological process made up the majority (25,528, 48.52%) followed by cellular component (17,165, 32.63%) and molecular function (9,917, 18.85%) (Figure 5). Under the biological process category, cellular process (4,696 unigenes, 18.40%) and metabolic process (3,726 unigenes, 14.60%) were prominently represented (Figure 5). In the category of cellular component, cell (5,884 unigenes) and cell part (5,243unigenes) represented the majorities of category (Figure 5). For the molecular function category, binding (4,223 unigenes) and catalytic activity (3,869 unigenes) was prominently represented (Figure 5).

**Figure 6:**
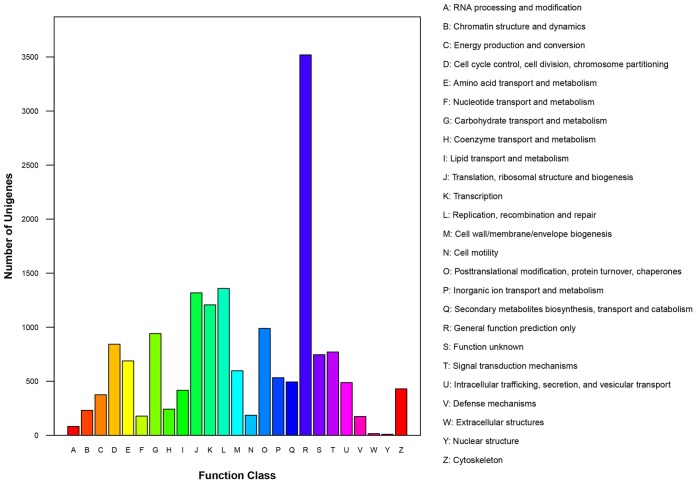
Histogram presentation of clusters of orthologous groups (COG) classification. Out of 30,427 nr hits, 9,009 sequences have a COG classification among the 25 categories.

The Cluster of Orthologous Groups (COG) is a database where the orthologous gene products were classified. All unigenes were aligned to the COG database to predict and classify possible functions [26]. Out of 30,427 nr hits, 9,009 sequences were assigned to the COG classifications (Figure 6). Among the 25 COG function categories, the cluster for General function prediction only (3,519, 20.90%) represented the largest group, followed by replication, recombination and repair (1,359, 8.07%) (Figure 6).

### Functional Classification by KEGG

The Kyoto Encyclopedia of Genes and Genomes (KEGG) Pathway database records the networks of molecular interactions in the cells, and their variants of them specific to particular organisms. In order to identify the biological pathways involved, the assembled unigenes were annotated with corresponding Enzyme commission (EC) numbers from BLASTX alignments against the KEGG database [27]. Firstly, based on a comparison against the KEGG database using BLASTX with an E-value cutoff of <10^−5^, out of the 116,885 unigenes, 19,611 (16.78%) had significant matches in the database and were assigned to 242 KEGG pathways. The pathways most represented by unique sequences were metabolic pathways (2,282 members), Huntington’s disease (683 members), purine metabolism (661 members), RNA transport (629 members), and regulation of actin cytoskeleton (306 members).

**Figure 7:**
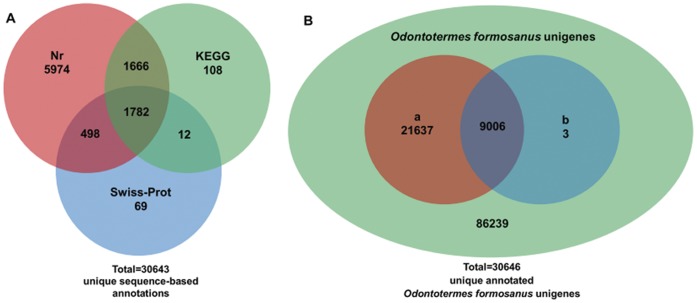
Distribution of similarity search results showed by Venn diagrams. (A) The number of unique sequence-based annotations is the sum of unique best BLASTX hits from the nr, Swiss-Prot and KEGG databases (E-value≤1.0E-5), respectively. The overlap regions among the three circles contain the number of unigenes that share BLASTX similarity with respective databases. (B) Number of all annotated *Odontotermes formosanus* unigenes is figured out based on the summation of both unique sequence-based annotations and unique domain-based annotations. The circle “a” and “b” indicate the two subsets of *O. formosanus* unigenes with sequence-based annotations and domain-based annotations, respectively.

Taken together, 30,643 unique sequence-based annotations had BLAST scores exceeding our threshold (≤1e-5) in nr, Swiss-Prot and KEGG databases (Figure 7A). The Venn diagram (Figure 7B) shows that an additional 3 unigenes were annotated by domain-based alignments. Overall, 30,646 unique sequence-based or domain-based annotations using the four selected public databases were assigned to *O. formosanus* unigenes (26.2%). Among them, 8,458 unigenes had hits in all four public databases with relatively defined functional annotations of the assembled unigenes (Table S2). These annotations provide a valuable resource for investigating specific processes, structures, functions, and pathways in caste differentiation.

### Protein Coding Region Prediction (CDS)

To further analyze unigene function at the protein level, we predicted the protein coding region (CDS) of all unigenes. First, we matched unigene sequences against protein databases by using BLASTX (E-value<0.00001) in the order: nr-Swissprot-KEGG-COG. Unigene sequences with hits in a database will not be included in the next round of search against another database. These BLAST results were used as information to extract CDS from unigene sequences and translate them into peptide sequences. In addition, BLAST results information is also used to train ESTScan [28], [29]. CDS of unigenes with no hit on BLAST search were predicted by ESTScan and then translated into peptide sequences. In total, 30,606 and 6,429 unigenes were predicted by using BLASTX and ESTScan, respectively. The histogram as seen in Figure S1 and Figure S2 shows the length distribution of CDS predicted from BLAST and ESTScan results. In general, as the sequence length increases, the number of CDS becomes gradually reduced. This is consistent with the results of unigene assembly.

### Frequency and Distribution of EST-SSRs in the Head Transcriptome

In total, 10,052 sequences containing 11,661 SSRs were predicted from 116,885 consensus sequences (Table S3). The EST-SSR frequency in the head transcriptome was 9.98%. The most abundant type of repeat motif was dinucleotide (39.66%), followed by trinucleotide (38.88%), tetranucleotide (16.57%), pentanucleotide (3.30%), and hexanucleotide (1.59%) repeat units (Table 2). The frequencies of EST-SSRs with different numbers of tandem repeats were calculated and shown in Table 2. The SSRs with six tandem repeats (21.14%) were the most common, followed by five tandem repeats (20.42%), seven tandem repeats (17.66%), and four tandem repeats (11.59%). The SSRs predicted in this study could lay a platform for better understanding the molecular ecology of *O. formosanus* as reported in the other termite species [30]. However, all the predicted SSRs need to be verified to exclude false positives and sequencing errors.

**Table 2:** Frequency of EST-SSRs in the head transcriptome of *Odontotermes formosanus*.

Motif length	Repeat numbers								Total	%
	4	5	6	7	8	9	10	>10		
**Di**	–	–	1,140	905	801	707	593	479	4,625	39.66
**Tri**	–	1,642	1,126	1,115	275	74	60	242	4,534	38.88
**Tetra**	935	679	171	29	29	22	8	59	1,932	16.57
**Penta**	280	43	14	7	5	6	2	28	385	3.3
**Hexa**	137	17	14	3	6	4	0	4	185	1.59
**Toatl**	1,352	2,381	2,465	2,059	1,116	813	663	812		
**%**	11.59	20.42	21.14	17.66	9.57	6.97	5.69	6.96		

### Putative Genes Involved in Caste Differentiation

The progress in molecular, genomic, and integrative biology have greatly improved understanding molecular basis underlying caste differentiation in termites [31]. From the current transcriptome database, we obtained seven putative genes with significant hits to 7 different genes known to be involved in termite caste differentiation by BLASTX analyses (Table 3). The previous RNAi analysis showed that the two genes (*hexamerin 1* and *2*) participate in the regulation of caste differentiation in *Reticulitermes flavipes* [1]. The gene, *Neofem2* coding for *β-glycosidase*, was necessary for the queen to suppress worker reproduction [4]. The gene, *Rf β-NAC-1* homologous to *bicaudal,* might affect the generalized soldier body plan [32]. In *R. flavipes*, multiple fat-body-related *CYP4* genes were differentially expressed in workers after juvenile hormone (JH) treatment [33]. The gene, *Nts19-1* which encodes putative homologues of the *geranylgeranyl diphosphate* (*GGPP*) *synthase* gene, is highly expressed exclusively in soldier head of *Nasutitermes takasagoensis* [34]. The head cDNAs analysis revealed that *Cox III* is differentially expressed between castes of *R. santonensis*, with lowest levels in the soldiers [35].

**Table 3:** Putative genes involved in castes differentiation.

Gene Annotation	Gene ID	Length (bp)	Subject ID	Species	E value
*hexamerin 1*	Unigene30435	374	BAG48838.1	*Reticulitermes speratus*	2E-50
*hexamerin 2* *	Unigene34583	2575	AAU20852.2	*Reticulitermes flavipes*	0
*β-glycosidase* *	Unigene34266	1238	AAL40863.1	*Rhyparobia maderae*	4E-76
*bicaudal D* *	Unigene55044	1072	EFA07458.1	*Tribolium castaneum*	1E-132
*CYP4U3v1*	CL6118.Contig1	1998	ABB86762.2	*Reticulitermes flavipes*	0
*GGPP synthase*	Unigene57705	526	BAJ79290.1	*Reticulitermes speratus*	4E-40
*cytochrome oxidase III*	Unigene41579	239	YP_002650710.1	*Dermatophagoides pteronyssinus*	6E-24

* denotes a gene selected for qPCR.

**Figure 8:**
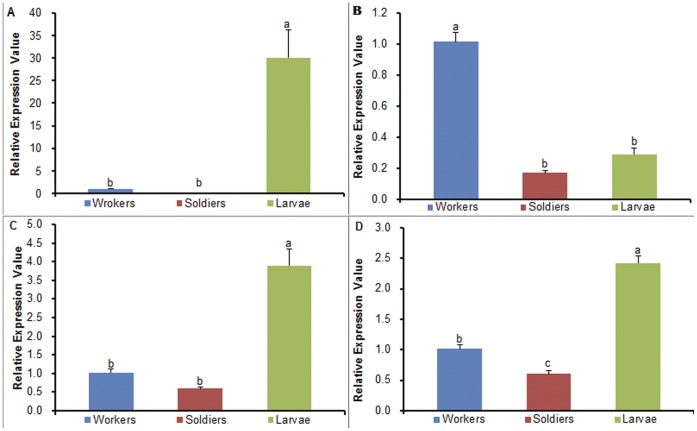
The qPCR analysis of putative genes involved in caste differentiation and aggression. The x-axis indicates three different castes. The y-axis indicates the relative expression value of uingene. (A) mRNA relative expression values for *hexamerin 2*. (B) mRNA relative expression values for *β-glycosidase*. (C) mRNA relative expression values for *bicaudal D*. (D) mRNA relative expression values for *Cyp6a20*. Letters above each bar denote significantly different groups. Significant differences were identified by a one-way ANOVA with means separated using Tukey’s HSD (P<0.05).

In this study, we selected three genes homologous to *hexamerin 2*, *β-glycosidase* and *bicaudal D* to analyze their expression differences among workers, soldiers and larvae of *O. formosanus* (Table S4), in order to detect whether the three genes are related to the caste differentiation of *O. formosanus*. The quantitative real-time PCR (qPCR) analysis showed that there was a significant difference in expression level of *hexamerin 2* among workers, soldiers and larvae (P<0.05). The *hexamerin 2* expression level in larvae was significantly higher than workers and soldiers, but there was no significant difference between workers and soldiers (Figure 8A). The two genes, *hexamerin 1* and *2*, have a “status-quo” presoldier-inhibitory function in workers [1]. In this study, the highest expression level of *hexamerin 2* in larvae suggests that most of larvae might develop into workers rather than soldiers.

The results indicated that there was a significant difference in expression level of *β-glycosidase* among workers, soldiers and larvae (P<0.05). The *β-glycosidase* expression level in workers was significantly higher than larvae and soldiers, but there was no significant difference between larvae and soldiers (Figure 8B). The gene, *Neofem2* coding for *β-glycosidase*, was highly overexpressed in female neotenics compared with workers in *C. secundus* [36]. Although the expression level of *β-glycosidase* in reproductives of *O. formosanus* was not analyzed in this study, our results suggest that the higher expression level of *β-glycosidase* in workers might be related to the function of breaking down polysaccharides [37].

Our results showed that there was a significant difference in expression level of *bicaudal D* among workers, soldiers and larvae (P<0.05). The *bicaudal D* expression level in larvae was significantly higher than workers and soldiers, but there was no significant difference between workers and soldiers (Figure 8C). In contrast, the expression level of *Rf β-NAC-1* homologous to *bicaudal* was the highest in soldiers of *R. flavipes,* indicating that *Rf β-NAC-1* in soldiers might influence the generalized soldier body plan [32]. However, our results suggest that *bicaudal D* might play an important role in larval development in *O. formosanus*.

### Putative Genes Involved in Aggression

Aggressive behavior is important for the survival and reproduction of many animal species [38]–[40], and is affected by genetic and environmental factors [41]. There is obvious interspecific and intercolonial aggression in termites, [42]. However, very little is known about molecular mechanisms underlying aggression in termites. From the current transcriptome database, we obtained six putative genes with significant hits to 6 different genes known to be involved in aggression by BLASTX analyses (Table 4). The gene *Cyp6a20* encoding a cytochrome P450, has been shown to modulate aggression in *Drosophila* [43], [44]. The drug-induced increases of *5-HT* in the brain increased *Drosophila* aggression [45], while the reduction of the neurotransmitter *octopamine* decreased *Drosophila* aggression [46]. The neurotransmitter *dopamine* also modulates aggressive behavior in *Drosophila* [47]. The inhibition of *MAOA* activity in mice leads to decreased aggression [48].

**Table 4:** Putative genes involved in aggression.

Gene Annotation	Gene ID	Length (bp)	Subject ID	Species	E value
*Cyp6a20* *	Unigene34391	2677	CP6A2_DROME	*Drosophila melanogaster*	3E-112
*dopamine transporter*	Unigene3655	398	SC6A2_MOUSE	*Mus musculus*	1E-25
*5-HT receptor*	CL523.Contig1	1439	GP119_RAT	*Rattus norvegicus*	4E-17
*5-HT transporter*	Unigene49370	1058	SC6A4_DROME	*Drosophila melanogaster*	0
*octopamine receptor*	Unigene25977	750	OCTB2_DROME	*Drosophila melanogaster*	4E-27
*monoamine oxidase A (MAOA)*	Unigene17133	438	BAB40325.1	*Canis lupus familiaris*	1E-19

* denotes a gene selected for qPCR.

In this study, we selected the gene homologous to *Cyp6a20* to analyze its expression differences among workers, soldiers and larvae of *O. formosanus* (Table S4), in order to detect whether this gene is involved in aggression regulation in *O. formosanus*. Our results showed that there was a significant difference in expression level of *Cyp6a20* among workers, soldiers and larvae (P<0.05). The *Cyp6a20* expression level in larvae was significantly higher than workers and soldiers, and the *Cyp6a20* expression level in workers was significantly higher than soldiers (Figure 8D). Additionally, our behavioral observations found that aggressiveness of soldiers are the highest among all the castes of *O. formosanus* [12]. The previous studies showed that the *Cyp6a20* expression levels might be negatively correlated with aggression [43]–[44]. Therefore, we suggest that *Cyp6a20* may be a candidate gene that downregulates aggression in *O. formosanus*.

### Conclusions

We have generated a comprehensive head transcriptome of *O. formosanus* using the Illumina sequencing. A single run produced more than 116,885 unigene sequences with 30,646 sequences with an above cut-off BLAST result. A total of 11,661 SSRs were predicted from the head transcriptome database. To our knowledge, this is the first attempt to characterize the complete head transcriptome of a higher termite using Illumina sequencing. Our study has changed the current status of lacking genetic information for *O. formosanus*, and has provided comprehensive sequence resources available for elucidating molecular mechanisms underlying caste differentiation and aggression in *O. formosanus*.

## Materials and Methods

### Sample Collection and Preparation

The *O. formosanus* colonies were collected from the three forests (Shizi, Yujia and Luojia) in Wuhan city, China. The three forests are not privately-owned or protected in any way, and *O. formosanus* is not endangered or protected in any way. Thus, no specific permissions are required for these locations/activities in this study. Healthy workers were selected from these colonies. We used scalpel to separate heads from bodies of workers. Then, head samples were immediately stored in liquid nitrogen for further processing.

### RNA Isolation, cDNA Library Construction and Illumina Sequencing

For Illumina sequencing, the total RNA of the head sample was extracted using TRIzol reagent (TaKaRa) according to the manufacturer’s protocol. The mixed RNA extract was subjected to Solexa sequencing analysis at the Beijing Genomics Institute (BGI; Shenzhen, China). RNA quality and quantity were verified using a NanoDrop 1000 spectrophotometer and an Agilent 2100 Bioanalyzer prior to further processing at BGI, and RNA integrity was confirmed with a number value of 8.6. The samples for transcriptome analysis were prepared using Illumina’s kit following manufacturer’s recommendations. Briefly, mRNA was purified from 44.4µg of total RNA using oligo (dT) magnetic beads. Fragmentation buffer was added for generation of short mRNA fragments. Taking these short fragments as templates, random hexamer-primer was used to synthesize the first-strand cDNA. The second-strand cDNA is synthesized using buffer, dNTPs, RNaseH and DNA polymerase I, respectively. Short fragments are purified with QiaQuick PCR extraction kit and resolved with EB buffer for end reparation and adding poly (A). After that, the short fragments were connected with sequencing adapters. And, after the agarose gel electrophoresis, the suitable fragments were selected for the PCR amplification as templates. At last, the library could be sequenced using Illumina HiSeq™ 2000.

### *De novo* Assembly of Sequencing Reads and Sequence Clustering

The cDNA library was sequenced on the Illumina sequencing platform. Image deconvolution and quality value calculations were performed using the Illumina GA pipeline 1.3. The raw reads were cleaned by removing adaptor sequences, empty reads and low quality sequences (reads with unknown sequences ‘N’). *De novo* transcriptome assembly was carried out with short reads assembling program – Trinity [21]. Trinity firstly combined reads with certain length of overlap to form longer fragments, which are called contigs. Then the reads were mapped back to contigs; with paired-end reads it was able to detect contigs from the same transcript as well as the distances between these contigs. Trinity connected the contigs, and gets sequences that cannot be extended on either end. Such sequences were defined as unigenes. When multiple samples from a same species were sequenced, unigenes from each sample’s assembly could be taken into further process of sequence splicing and redundancy removing with sequence clustering software to acquire non-redundant unigenes as long as possible.

### Analysis of Illumina Sequencing Results

Unigene sequences were firstly aligned by BLASTX to databases like nr, Swiss-Prot, KEGG and COG (E-value <0.00001), retrieving proteins with the highest sequence similarity with the given unigenes along with their protein functional annotations, the results about this were included in the folder annotation. With nr annotation, we used Blast2GO program to get GO annotation of unigenes. After getting GO annotation for every unigene [24], we used WEGO software to do GO functional classification for all unigenes and to understand the distribution of gene functions of the species from the macro level [25]. With the help of KEGG database, we could further study genes’ biological complex behaviors, and by KEGG annotation we could get pathway annotation for unigenes.

When predicting the CDS, we first aligned unigenes to nr, then Swiss-Prot, then KEGG, and finally COG. Unigenes aligned to a higher priority database will not be aligned to lower priority database. The alignments end when all alignments were finished. Proteins with highest ranks in BLAST results were taken to decide the coding region sequences of unigenes, and then the coding region sequences were translated into amino sequences with the standard codon table. So both the nucleotide sequences (5′–3′) and amino sequences of the unigene coding region were acquired. Unigenes that cannot be aligned to any database are scanned by ESTScan, producing nucleotide sequence (5′–3′) direction and amino sequence of the predicted coding region [28].

### EST-SSR Detection

Putative SSR markers were predicted among the 116,885 unigenes using Serafer [49]. The parameters were adjusted for identification of perfect di-, tri-, tetra-, penta-, and hexanucleotide motifs with a minimum of 6, 5, 4, 4, and 4 repeats, respectively. Mononucleotide repeats were ignored because it was difficult to distinguish genuine mononucleotide repeats from polyadenylation products and single nucleotide stretch errors generated by sequencing.

### Gene Mining and Quantitative Real Time PCR

Total RNA was extracted from heads of workers, soldiers and larvae using TRIzol following the manufacturer’s protocol. Approximately 1 µg of DNase I-treated total RNA was converted into single-stranded cDNA using a PrimeScript RT regent reagent Kit (perfect real time) (TaKaRa, Dalian, China). The cDNA products were then diluted 80-fold with deionized water before use as a template in real-time PCR. The quantitative reaction was performed on a My IQ™ 2 Two color Real-time PCR Detection System (Bio-Rad, USA) using SYBR Premix Ex Taq™ II (TaKaRa, Dalian, China). The reaction mixture (20 µL) contained 2×SYBR Premix Ex Taq™ II 10 µL, 0.4 µM each of the forward and reverse primers, and 2 µL of template cDNA. PCR amplification was performed under the following conditions: 95°C for 30 s, followed by 40 cycles of 95°C for 5 s and 60°C for 30 s, at last by 55°C for 30 s. The expression of four interesting genes were normalized against an internal reference gene, *β-actin*. Primers were designed using Beacon Designer 7.7 software (primer sequences upon request) (Table S4). For caste-specific expression assay, expression in workers was used as the calibrator for each gene. The relative gene expression was calculated using the 2^−ΔΔ^Ct method [50]. All qPCR were repeated in three biological and three technical replications. Differences in expression level of the four genes among workers, soldiers and larvae were tested for significance by a one-way ANOVA with means separated using Tukey’s HSD (SPSS Inc., 1989–2002).

### Data Deposition

The Illumina sequencing reads of worker heads of *O. formosanus* were submitted to NCBI Sequence Read Archive under the accession number of SRA055431.

## Supporting Information

Figure S1 **Length distribution of CDS predicted from BLAST.** The x-axis shows read size and the y-axis shows the number of reads for each given size.
(TIF)

Figure S2 **Length distribution of CDS predicted from ESTScan.** The x-axis shows read size and the y-axis shows the number of reads for each given size.
(TIF)

Table S1 **Top BLAST hits from NCBI nr database.** BLAST results against the NCBI nr database for all the distinct sequences with a cut-off E-value above 10^−5^ are shown.
(XLSX)

Table S2 **BLAST hits from the four databases (nr, KEGG, COG, Swiss-Prot).**
(XLSX)

Table S3 **Predicted EST-SSRs in the head transcriptome of** ***Odontotermes formosanus*** **.**
(XLSX)

Table S4 **Interesting gene ID in the head transcriptome and primers used for qPCR.**
(DOC)
