# Supplementary figures and images for: Characterization of Head Transcriptome and Analysis of Gene Expression Involved in Caste Differentiation and Aggression in Odontotermes formosanus (Shiraki)
Source: PLoS One. 2012 Nov 29;7(11):e50383. doi: 10.1371/journal.pone.0050383 (PMC3510212; doi:10.1371/journal.pone.0050383)

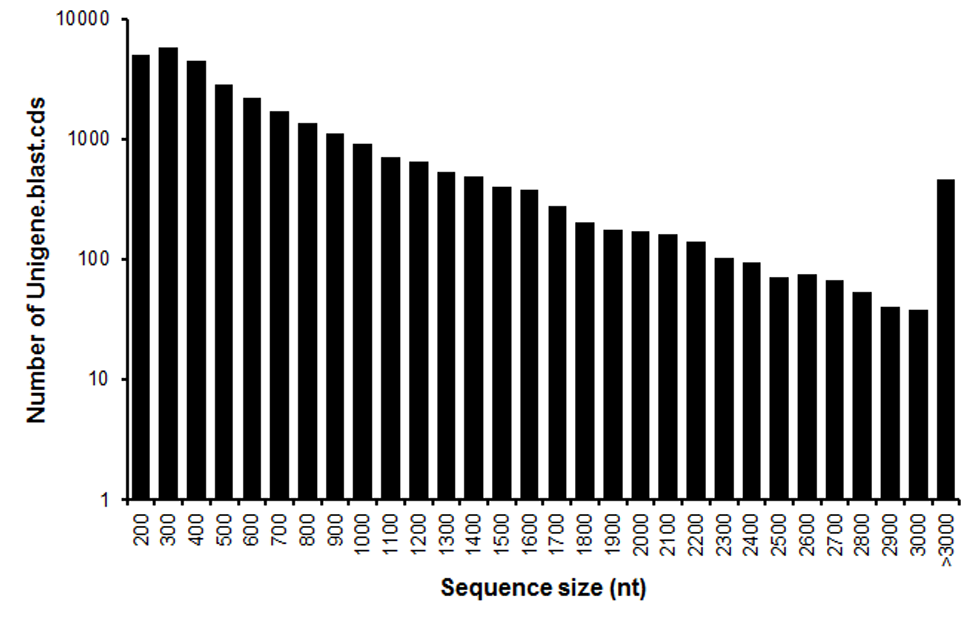

Supplement: Figure S1 — Length distribution of CDS predicted from BLAST. The x-axis shows read size and the y-axis shows the number of reads for each given size. (TIF) [file pone.0050383.s001.tif]

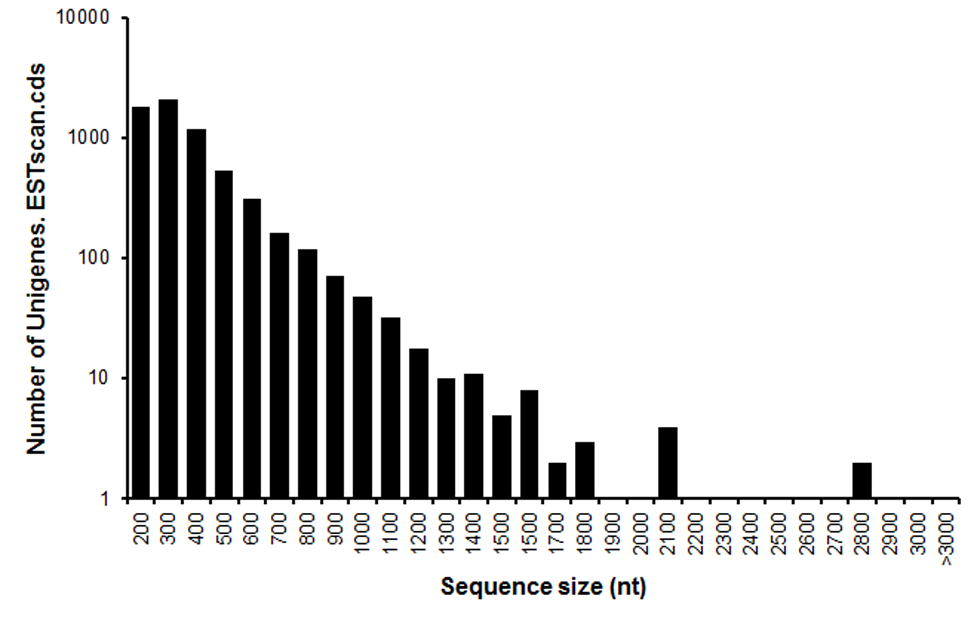

Supplement: Figure S2 — Length distribution of CDS predicted from ESTScan. The x-axis shows read size and the y-axis shows the number of reads for each given size. (TIF) [file pone.0050383.s002.tif]
