# Supplementary material for: Characterization of Head Transcriptome and Analysis of Gene Expression Involved in Caste Differentiation and Aggression in Odontotermes formosanus (Shiraki)
Source: PLoS One. 2012 Nov 29;7(11):e50383. doi: 10.1371/journal.pone.0050383 (PMC3510212; doi:10.1371/journal.pone.0050383)
Supplement: Table S4 — Interesting gene ID in the head transcriptome and primers used for qPCR. (DOC) [file pone.0050383.s006.doc]

**Table S4** Interesting gene ID in the head transcriptome and primers used for qPCR.

| **Gene ID** | **Primer name** | **(5′→3′) nucleotide sequence** |
| --- | --- | --- |
| **Unigene34583** | *Hexamerin 2 F* | GGTCTCTTATCTGCTCTATCA |
|  | *Hexamerin 2 R* | TCTGCTATACTTCCTTCCTG |
| **Unigene34266** | *β-glycosidase F* | GGTCTCTTATCTGCTCTATCA |
|  | *β-glycosidase R* | TCTGCTATACTTCCTTCCTG |
| **Unigene55044** | *bicaudal D F* | GGTCTCTTATCTGCTCTATCA |
|  | *bicaudal D R* | TCTGCTATACTTCCTTCCTG |
| **Unigene34391** | *Cyp6a20 F* | GGTCTCTTATCTGCTCTATCA |
|  | *Cyp6a20 R* | TCTGCTATACTTCCTTCCTG |
| **CL8763.Contig1** | *β-actin F* | GGTCTCTTATCTGCTCTATCA |
|  | *β-actin R* | TCTGCTATACTTCCTTCCTG |
